# Modulation of double-negative T cells by Huang-Lian-Jie-Du Decoction attenuates neuroinflammation in ischemic stroke: insights from single-cell transcriptomics

**DOI:** 10.3389/fimmu.2025.1537277

**Published:** 2025-02-13

**Authors:** Kai Wang, Zijin Sun, Qi Shao, Zhaoyi Wang, Haojia Zhang, Yuanhua Li, Jingmei Ming, Wenjing Zhang, Tieshan Wang, Yan Zhao, Qingguo Wang, Fafeng Cheng

**Affiliations:** Chinese Medicine College, Beijing University of Chinese Medicine, Beijing, China

**Keywords:** ischemic stroke, double-negative T cells, Huang-Lian-Jie-Du Decoction, immune modulation, neuroprotection

## Abstract

**Introduction:**

Ischemic stroke (IS) represents a significant global health challenge, characterized by elevated morbidity and mortality rates, largely driven by inflammatory responses. Double-negative T cells (DNTs), a distinct subset of T cells lacking both CD4 and CD8 markers, have been implicated in the pathogenesis of IS, exhibiting potentially dual roles. However, the precise functional contributions of DNTs in this context remain poorly understood.

**Methods:**

In this study, we investigated the role of DNTs during the acute phase of IS and assessed the influence of Huang-Lian-Jie-Du Decoction (HLJD), a traditional Chinese medicinal formula, on these cells. Using single-cell transcriptomics, we identified two distinct subtypes of DNTs: an activated, cytotoxic phenotype (Kill+) and a resting, immunosuppressive phenotype (Kill-).

**Results:**

Our findings indicate that HLJD treatment modulates the balance between these DNT subtypes, specifically reducing the proportion of cytotoxic DNTs while promoting an increase in immunosuppressive DNTs. This shift was associated with a reduction in immune cell infiltration and inflammation within the brain tissue, potentially mitigating neuronal damage.

**Discussion:**

These results suggest that HLJD exerts neuroprotective effects in IS by modulating the activity and distribution of DNT cells, offering valuable insights into the therapeutic potential of traditional Chinese medicine for the treatment of IS. Further studies are required to elucidate the mechanisms underlying DNT-mediated immune responses in IS and to explore the broader applications of HLJD in other neuroinflammatory conditions.

## 1 Introduction

Stroke is a highly detrimental global health concern, ranking as the second leading cause of death and exhibiting an alarmingly high disability rate, thus imposing severe consequences on individuals and considerable burdens on national healthcare systems (1). Stroke can be categorized into hemorrhagic and ischemic types, with acute ischemic stroke (AIS) being the most common, accounting for approximately 60% to 80% of all stroke cases. Moreover, neuroinflammatory damage caused by immune cells is increasingly recognized as an important factor leading to AIS injury.

Double-negative T cells (DNTs) are a subset of T cells lacking the surface markers CD4 and CD8, hence the designation “double-negative.” These cells are generated during thymic development and represent an atypical T cell population. Although relatively scarce, DNT cells play a critical role in maintaining immune homeostasis, modulating immune responses and regulating inflammation levels. They have gained increasing attention in the regulation of immune tolerance and in the management of autoimmune diseases. Research has extensively explored their roles and applications in diseases such as systemic lupus erythematosus, non-alcoholic fatty liver disease, and various cancers (2–6).

In recent years, attention has increasingly turned toward ischemic stroke (IS) (7). However, our understanding of DNTs remains limited, with some studies even reporting contradictory findings (8, 9). Consequently, elucidating the role and specific functions of DNTs in IS is essential to advance our knowledge and improve therapeutic strategies.

Huang-Lian-Jie-Du Decoction (HLJD) is a traditional Chinese medicine formula composed of Coptis chinensis, Scutellaria baicalensis, Phellodendron amurense, and Gardenia jasminoides, known in traditional Chinese medicine for its “heat-clearing and detoxifying” properties (10–13). HLJD has demonstrated neuroprotective, anti-neuroinflammatory effects and immunomodulation in neurological disorders, including ischemic stroke (IS). However, previous studies primarily focused on broad-spectrum neuroinflammation or the resident microglia within the brain, with limited exploration of the role of peripheral immune cells.

In this study, we utilize single-cell transcriptomics to investigate the specific role of DN T cells (DNTs) during the acute phase of stroke, clarifying their functional contributions. Furthermore, we examine the specific effects and regulatory mechanisms of HLJD on these cells, aiming to provide new insights for future clinical therapies.

## 2 Method

### 2.1 Design of the animal experiments

Eight-week-old male C57BL/6J mice, weighing 23 ± 2g, were sourced from Beijing Sibeifu Experimental Animal Technology Co., Ltd. The animals were housed under specific pathogen-free (SPF) conditions at a controlled facility at Beijing University of Chinese Medicine. The environment was maintained at 25 ± 1°C with 55 ± 10% relative humidity, on a 12-hour light/dark cycle, and free from noise disturbances.

All animal procedures were conducted following ethical guidelines, with approval from the Animal Ethics Committee of Beijing University of Chinese Medicine (Approval number: BUCM-2024011901-1036). The experimental protocols adhered strictly to animal welfare standards.

Mice were randomly divided into three groups using a random number table: the sham surgery group, the model group, and the HLJD treatment group.

Following a one-week acclimatization period, intragastric administration was initiated. Mice in the sham surgery and model groups received physiological saline, while the HLJD group was administered doses equivalent to the standard daily human dosage (based on a 60 kg adult) of freeze-dried HLJD powder.

To evaluate the therapeutic potential of HLJD for acute ischemic stroke, each mouse received a dose of 0.2 mL/10g of body weight daily for five consecutive days. On the sixth day, following the final administration, the transient middle cerebral artery occlusion (tMCAO) model was induced in the animals to mimic ischemic stroke conditions.

### 2.2 Neurobehavioral evaluation

Neurobehavioral assessments were conducted 24 hours after embolus insertion into the middle cerebral artery, using the Longa 5-point scale to evaluate the severity of neurological deficits. Mice were placed on a flat, unobstructed platform for assessment. Higher scores on the Longa scale indicated more severe neurological impairment.

### 2.3 TTC staining

Twenty-four hours after embolus insertion, mice were euthanized, and brain tissues were collected for 2,3,5-triphenyltetrazolium chloride (TTC) staining. This staining method enables the visualization of infarcted brain regions, differentiating viable from non-viable tissue.

### 2.4 Cerebral blood flow measurement

To assess cortical blood flow, mice were anesthetized with isoflurane and positioned in a stereotaxic apparatus. After disinfecting the head, a scalp incision was made to expose the skull area between the coronal suture and lambda. Laser speckle imaging was then employed to measure cerebral blood flow in the cortical region, providing quantitative data on blood flow changes.

### 2.5 Histological analysis with HE and Nissl staining

Brain tissues from the mice were fixed in 4% paraformaldehyde overnight and subsequently embedded in paraffin. Paraffin-embedded sections were stained with hematoxylin and eosin (HE) and Nissl staining solutions to assess structural and cellular changes in brain tissue, aiding in the evaluation of morphological alterations.

### 2.6 Single cell sample preparation

Peripheral blood and brain tissues were collected from the mice and preprocessed.

For peripheral blood, samples were obtained via retro-orbital bleeding. Anti-ter-119 magnetic beads and anti-dead cell magnetic beads were used to remove red blood cells and dead cells. After extraction, red blood cells were lysed with a lysis buffer to further eliminate red blood cells and dead cells. Cell count and viability were assessed using CountSTARS.

For brain tissue, after blood collection, the mice were perfused with autoMACS Rinsing Solution to flush out as much intracerebral blood as possible. Once the outflow fluid was clear, the mice were decapitated, and the ischemic hemisphere was isolated. The tissue was then transferred to a C-tube containing a mixture of enzymes, and dissociation was performed using the gentleMACS Octo Dissociator. After dissociation, cell debris was removed, and red blood cells were lysed. Flow cytometry was performed using CD47, CD106, and CD304 antibodies for incubation, followed by negative selection to retain as many immune cells as possible. Finally, cell count and viability were determined using CountSTARS.

The Chromium Next GEM Single Cell 3’ Kit was employed to generate single-cell oil-in-water droplets, followed by cDNA synthesis and library construction for single-cell transcriptomics. After quality control of the libraries, paired-end sequencing was conducted on the Illumina NovaSeq 6000 platform with a 2x150 bp read length.

After sequencing, the raw data from the Illumina platform were processed using Cell Ranger software (version cellranger-7.1.0; 10X Genomics), converting BCL files into FASTQ files. The Cell Ranger Count command quantified cells and genes, aligning raw reads to the mm10 reference genome using the built-in STAR aligner. Quality control metrics such as the number of high-quality cells, gene count, genome alignment rate, and the UMI (Unique Molecular Identifier) matrix were obtained based on the alignment results.

### 2.7 scRNA-seq enrichment analysis

Using five single-cell scoring methods—AUCell, UCell, singscore, ssGSEA, GSVA and Add—we performed enrichment scoring for the target gene set, resulting in a total enrichment score (14–16).

### 2.8 Transcription factor analysis

We performed transcription factor analysis using pyscenic to infer transcription factor activity using gene expression matrices (17).

### 2.9 Metabolic flux analysis

scFEA was applied to evaluate metabolite abundance based on scRNA-seq data (18).

### 2.10 Cell trajectory analysis

To understand the differentiation trajectories of cells, we utilized Monocle2 and Monocle3 software for analysis. To arrange cells along this inferred trajectory, we designated a starting point for the differentiation pathway. Finally, using the orderCells function, we sequentially positioned all cells along the established trajectory. This methodology allows us to visualize and analyze the progression of cellular differentiation within the dataset.

### 2.11 Cell-cell communication

The CellChat package was utilized for quantitatively inferring and analyzing cellular interactions from scRNA-seq data (19).

### 2.12 Statistical analysis

All data in this study were analyzed by using Prism 7 software. One-way analysis of variance (ANOVA) was performed to identify differences among groups. Data are presented as the means ± SDs. A P value < 0.05 was regarded as being significant.

## 3 Results

### 3.1 HLJD reduces neurological deficits and ischemic damage in MCAO model

**Figure 1:**
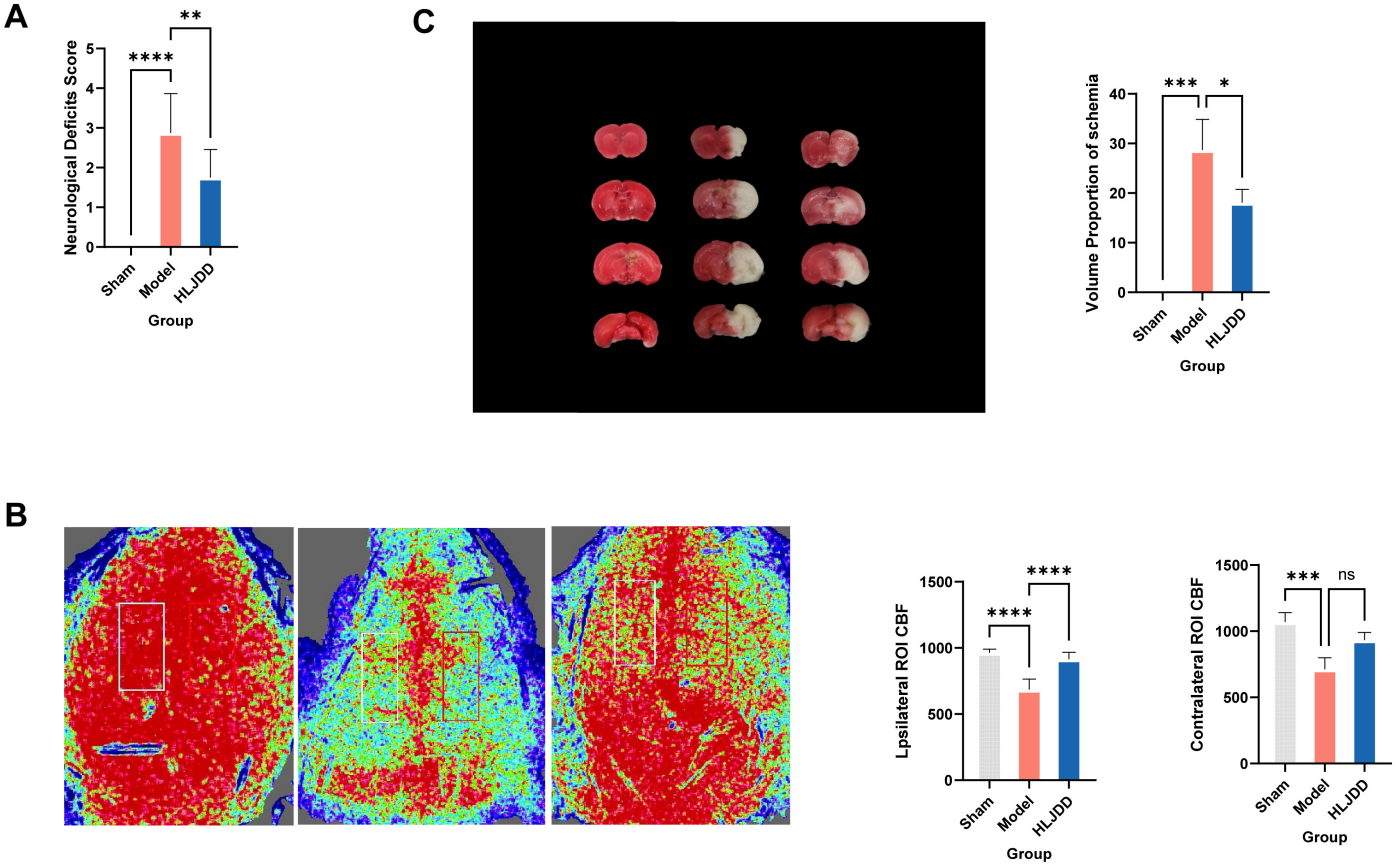
HLJD alleviates infarct area, neurological dysfunction, cerebral microvascular perfusion of mice after ischemic stroke. **(A)** The nervous system scores of each group 24 h after cerebral ischemia-reperfusion injury. **(B)** Representative images and statistical results of laser speckle analysis of cerebral blood perfusion. **(C)** Cerebral infarction volume after TTC staining in rats with cerebral ischemia-reperfusion injury. Compared with the model group,*p < 0.05, **p < 0.01, ***p < 0.001, ****p < 0.0001 (*n* = 6). NS, No significance.

We utilized neurological scoring, laser speckle imaging, and TTC staining to evaluate the severity of injury in the MCAO model and the therapeutic effects of HLJD. These methods provided insights from three perspectives: neurological deficit severity, real-time cerebral blood flow, and ischemic brain volume. The results clearly demonstrated that, following model induction, the mice exhibited neurological impairment(**Figure 1A**), with a marked decrease in real-time blood flow on the affected and ischemic sides compared to the control group, alongside the presence of severe ischemic lesions in brain tissue (**Figures 1B, C**). However, treatment with HLJD partially reversed these trends, indicating its potential therapeutic effects in ischemic stroke (IS).

### 3.2 HLJD preserves neuronal structure and mitigates ischemic damage in HE and Nissl staining analyses

**Figure 2:**
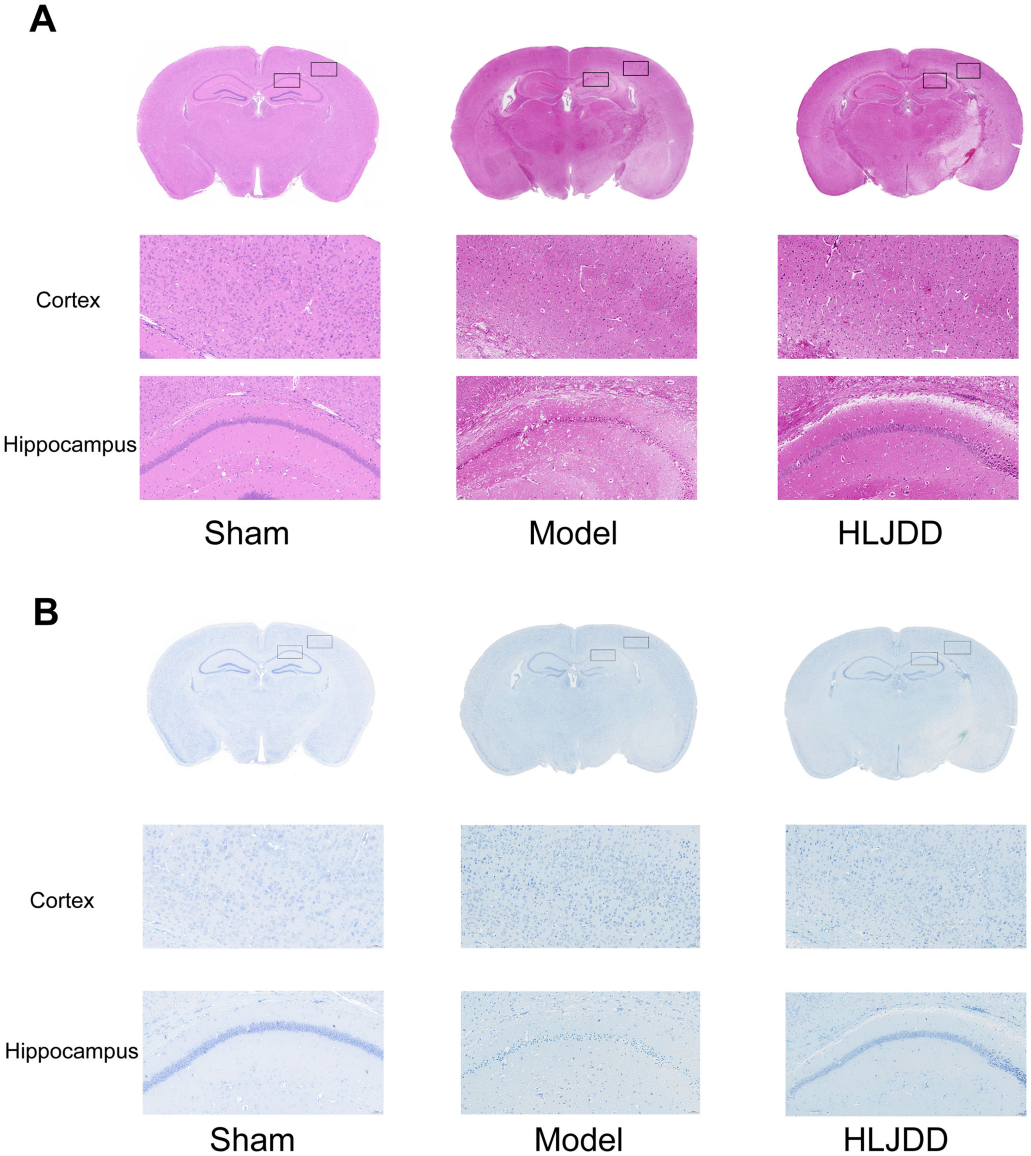
The histopathological changes of cortex and hippocampal tissue were observed by HE staining and Nissl staining (*n* = 5). **(A)** The cortical and hippocampal tissues of mice in each group were enlarged by 20 times after HE staining. **(B)** The cortical and hippocampal tissues of each group of mice were enlarged by 20 times after Nissl staining.

According to the HE staining results, compared to the normal group, the model group displayed unclear cortical layers, enlarged intercellular spaces, and signs of typical ischemic damage in neuronal cells, including cell body swelling (cytoplasmic vacuolization), nuclear shrinkage, and chromatin marginalization. In more severely affected regions, extensive necrosis and complete structural disintegration were observed, indicating significant pathological alterations (**Figure 2A**).

The Nissl staining further highlighted marked morphological changes in the affected brain regions. Many neurons exhibited pale Nissl bodies, reduced cell body size, and impaired cellular function. Additionally, neuronal dendrites appeared fragmented or shortened, suggesting structural damage. In the ischemic core, a substantial loss of the typical deep blue Nissl staining was observed, indicating necrosis or severe damage in neuronal cells (**Figure 2B**).

Following treatment with HLJD, these injuries showed varying degrees of improvement, supporting the neuroprotective effects of HLJD in preserving neuronal structure and function.

### 3.3 HLJD modulates immune cell composition and restores balance in ischemic stroke model

Based on previous research findings, HLJD likely exerts its protective effects against ischemic stroke (IS) damage by inhibiting inflammation-induced injury. To further investigate the effector cells and specific mechanisms involved, we conducted single-cell sequencing. To maximize the yield of immune cells for analysis, we used flow cytometry to remove certain non-immune cell types, enriching the immune cell population.

**Figure 3:**
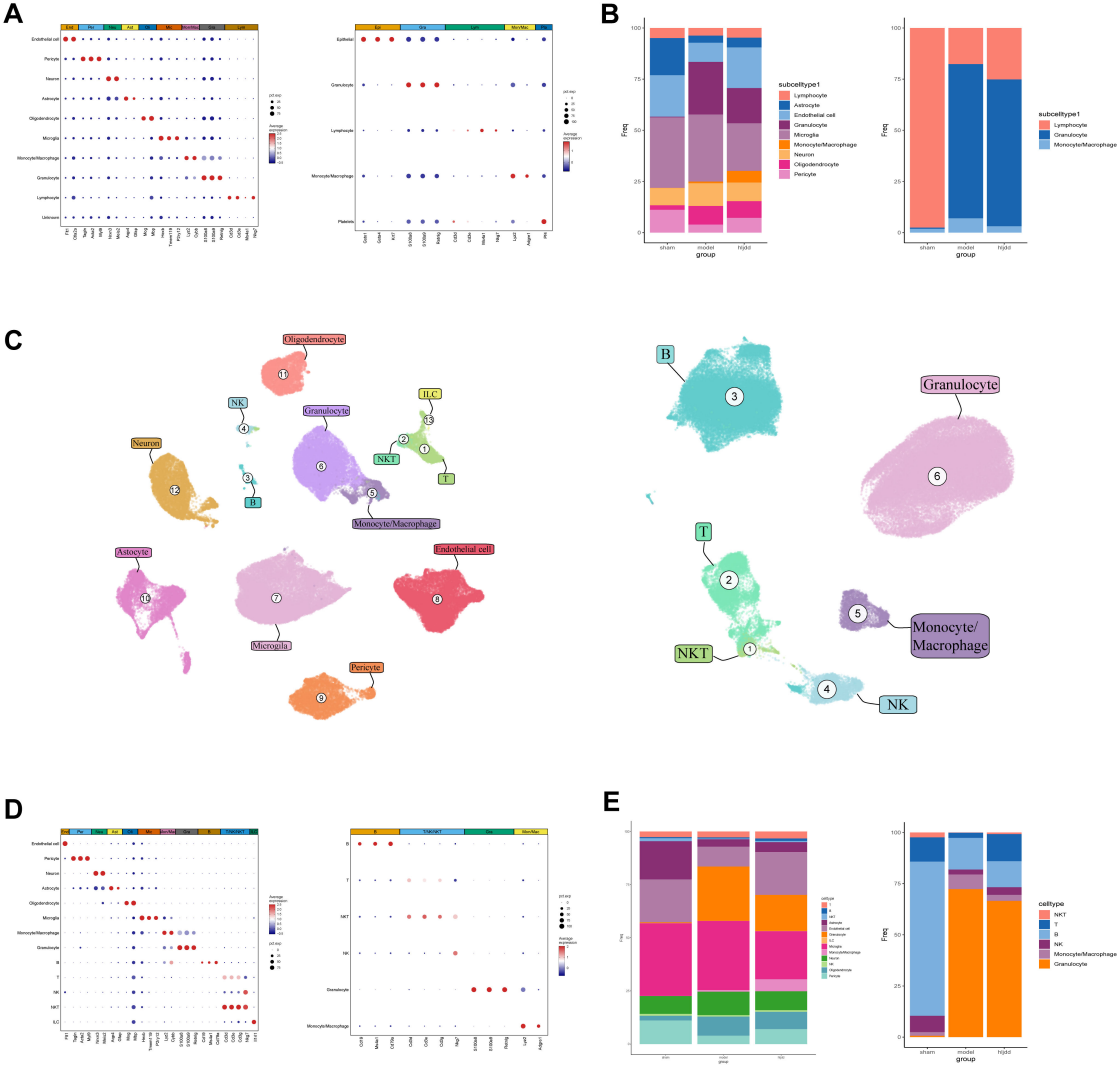
The landscape of monocytes in the brain and peripheral blood. **(A)** Brain and peripheral blood cell types and their corresponding markers. **(B)** Brain to peripheral blood cell ratio. **(C)** UMAP dimensionality reduction demonstration of brain and peripheral blood cells. **(D)** Secondary annotation of cellular composition of brain and peripheral blood with MARKER. **(E)** Stacked bar graphs show the proportion of major cell types in brain and blood after secondary annotation, with lymphocytes further subdivided.

Our single-cell analysis results revealed that peripheral blood cells could be categorized, based on specific markers (**Figure 3A**), into major groups such as myeloid cells (including neutrophils, monocytes/macrophages), lymphocytes (T cells, B cells, NK cells), and other cells (platelets, shed endothelial cells). In the central nervous system (CNS), cell types were distinguished into neurons, glial cells, vascular cells, and immune cells (**Figure 3B**). To accurately observe T cell dynamics, we performed quality control and filtering on our data, removing low-quality cells and cell types with minimal interaction, which were then excluded from subsequent analyses (**Figure 3C**).

Before stroke induction, lymphocytes dominated the immune cell composition in both peripheral blood and CNS-infiltrated cells. However, after stroke modeling, neutrophils overwhelmingly dominated the cell population. Following HLJD administration, this shift showed a partial reversal, indicating some recovery in cell composition balance.

We further refined the lymphocyte population into T cells, B cells, NK cells, NKT cells, and ILCs, finding that B cells displayed the most significant proportional change, followed by T cells (**Figures 3D, E**).

### 3.4 HLJD alters T cell dynamics and transcriptional activity in acute stroke phase

**Figure 4:**
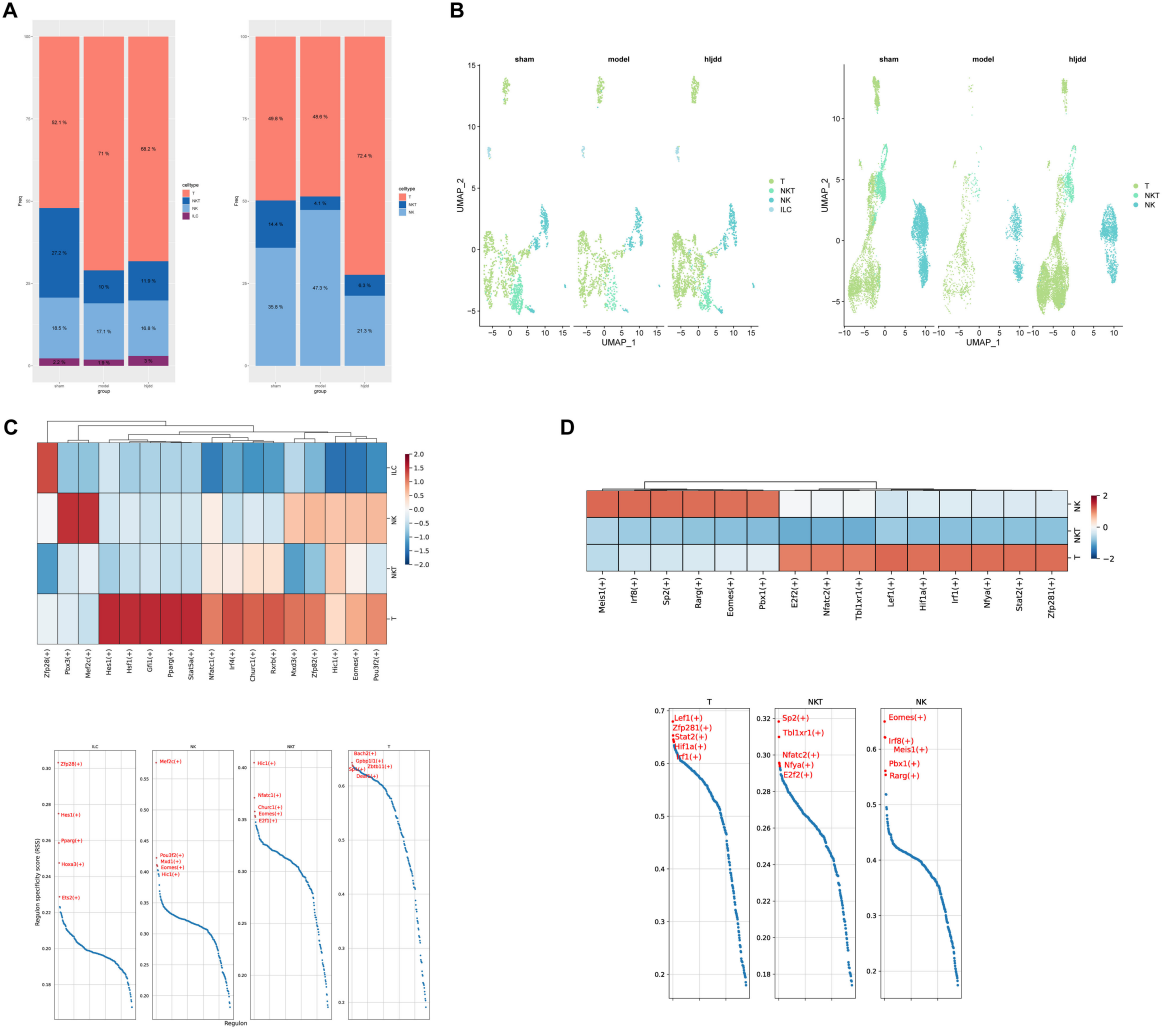
Analysis of killer lymphocytes. **(A)** The proportion of killer lymphocytes in brain and peripheral blood. **(B)** UMAP display of lethal lymphocytes in brain and blood. **(C)** Heat map of killer lymphocytes in the brain and presentation of the top five transcription factors. **(D)** Heat map of killer lymphocytes in blood and presentation of top five transcription factors.

In this study, we focused primarily on the acute phase of stroke, a period during which inflammation plays a central role (**Figures 4A, B**). Consequently, we isolated lymphocyte subtypes with cytotoxic and inflammatory regulatory functions for subsequent analyses. Among CNS-infiltrating cells, T cells and NKT cells showed the most substantial changes, whereas in the peripheral blood, T cells and NK cells exhibited significant variation. Interestingly, within brain tissue, T cells increased significantly following stroke induction. In contrast, in peripheral blood, the overall proportion of T cells appeared largely unaffected by the stroke itself; however, T cell counts in the peripheral blood rose markedly after HLJD treatment, showing an inverse trend compared to T cell dynamics within the brain.

To investigate the potential roles of these lymphocyte subsets, we performed transcription factor analysis using pySCENIC (**Figures 4C, D**). This analysis revealed that T cells within the broader cytotoxic lymphocyte category were the most transcriptionally active, suggesting they may play a critical role in the immune response during the acute phase of stroke.

### 3.5 HLJD promotes CNS migration of double-negative T cells in ischemic stroke

**Figure 5:**
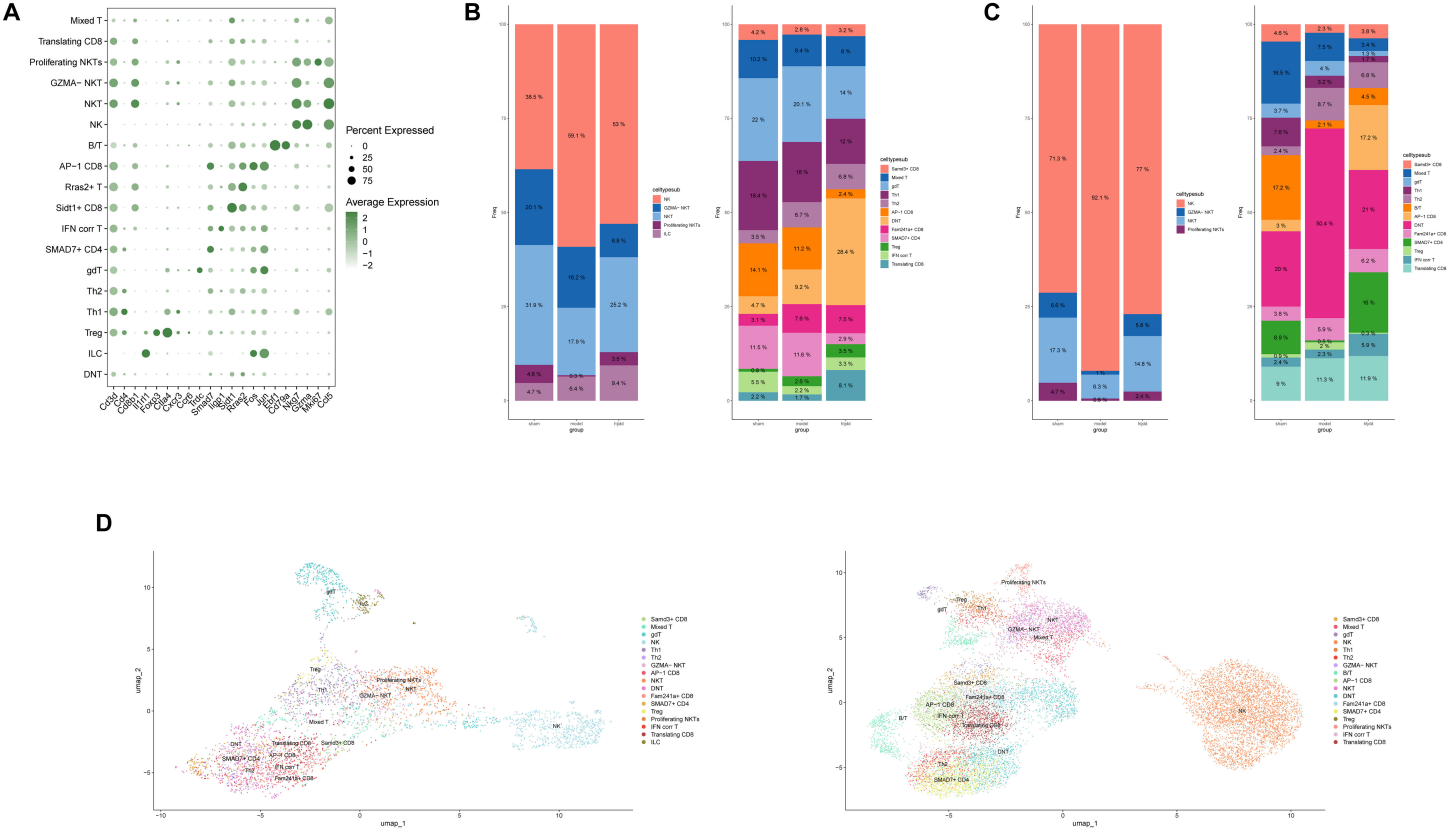
Immune landscape of killer lymphocytes in ischemic stroke. **(A)** Signature genes for a subset of killer lymphocytes. **(B)** The bar chart shows the ratio of killer lymphocytes subsets in the brain. **(C)** The bar chart shows the ratio of killer lymphocytes subsets in the blood. **(D)** Annotated UMAP colored by killer lymphocytes subsets.

Based on these findings, it is evident that T cells play a significant role in both the onset of stroke and the regulatory effects of Huang-Lian-Jie-Du Decoction (HLJD). However, cytotoxic lymphocytes, particularly T cells, are highly heterogeneous. To further clarify this heterogeneity, we subdivided these cells based on common markers and identified specific markers from our analysis (**Figure 5A**). Notably, following stroke induction, the population of double-negative T cells (DNTs) (CD3+, CD4-, CD8-, NKG7-) showed significant variation. Interestingly, after HLJD treatment, the number of DNTs increased in the CNS while decreasing in peripheral blood. This observation suggests that HLJD may promote the migration of DNTs from the periphery into the brain (**Figures 5B–D**).

### 3.6 Distinct metabolic and transcriptional profiles of DNT cells highlight unique role in stroke modulation by HLJD

**Figure 6:**
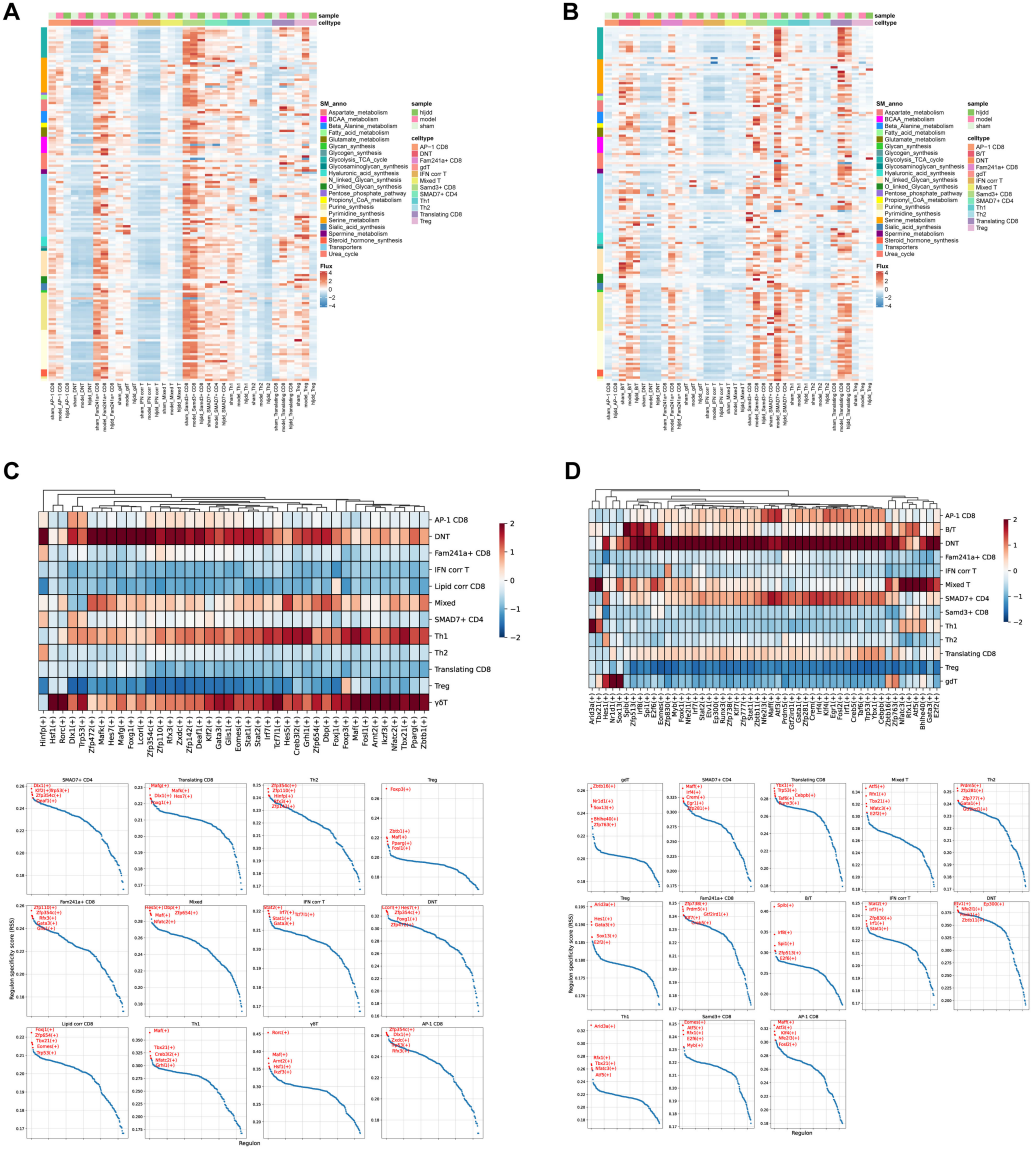
Exploration of T cell subset characteristics. **(A)** Heat map of differentially expressed metabolic pathways of each T cell subset in the brain identified by scFEA. **(B)** Heat map of differentially expressed metabolic pathways of each T cell subset in the blood identified by scFEA. **(C)** T cell subset transcription factor activity in the brain is shown against the top five transcription factors. **(D)** T cell subset transcription factor activity in the blood is shown against the top five transcription factors.

Subsequently, we conducted a detailed analysis of T cell subpopulations to investigate their metabolic states and transcription factor activity (**Figure 6**). As anticipated, nearly all T cell subsets exhibited elevated metabolic levels post-stroke, correlating with functional activation and increased energy consumption. However, following HLJD treatment, metabolic levels decreased to varying extents, suggesting that HLJD may inhibit T cell activation, thereby potentially reducing inflammation-related damage during the acute phase of stroke.

Interestingly, DNT cells displayed a relatively low metabolic level throughout, with only a few metabolic pathways showing modest increases post-stroke. In contrast, transcription factor analysis revealed that DNTs maintained high transcriptional activity compared to other T cell subtypes. This seemingly paradoxical finding highlights the unique nature of DNTs, prompting further investigation into their specific role and mechanisms.

### 3.7 HLJD modulates DNT cell subtypes in ischemic stroke, reducing inflammatory damage by inhibiting cytotoxic activation

**Figure 7:**
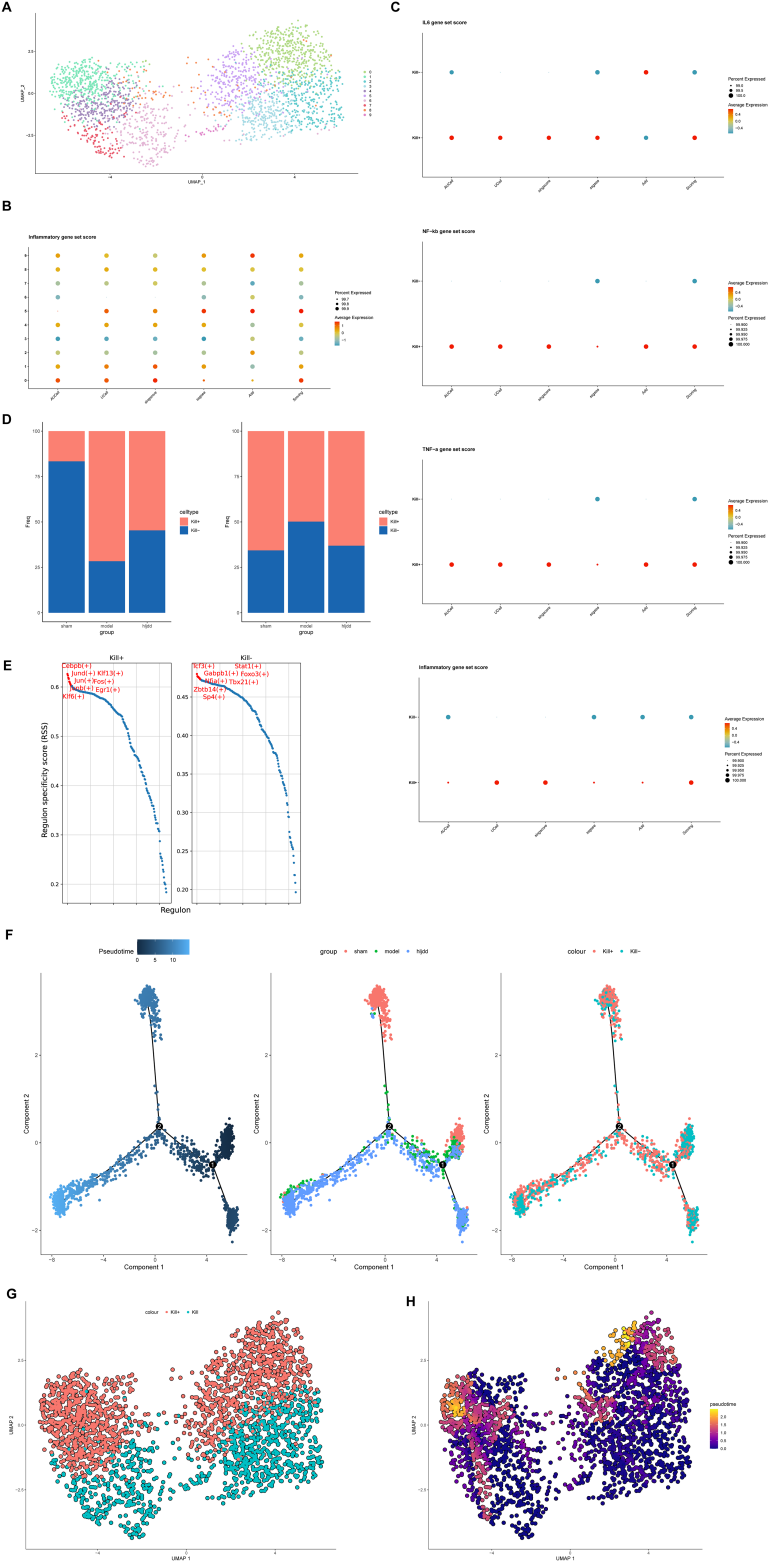
DNT reanalysis. **(A)** DNT can be divided into two categories by UMAP dimensionality reduction. **(B)** The scoring based on the inflammatory gene set can classify DNT into two categories. **(C)** Scores of multiple inflammatory pathway/phenotype gene sets between Kill+ DNT and Kill- DNT. **(D)** Transcriptional factor activity levels and top five transcription factors of Kill+ DNT and Kill- DNT. **(E)** The ratio of Kill+DNT and Kill-DNT in sham-operated group, model group and HLJD group. **(F)** Quasitemporal trajectories of Kill+DNT versus Kill-DNT calculated by Monocle2. **(G)** DNT cell distribution. **(H)** Quasitemporal trajectories of Kill+DNT versus Kill-DNT calculated by Monocle3 (Brighter colors indicate a higher degree of differentiation.).

The preceding results highlight the distinct and significant role of DNT cells in ischemic stroke (IS). However, DNT function within IS remains unclear and is a subject of diverse interpretations. To further investigate their role, we performed single-cell analysis on DNT cells. First, we conducted re-dimensional reduction, which showed that DNT cells could be unsupervisedly clustered into two main groups on the UMAP plot (**Figure 7A**). To explore the inflammatory phenotypes of these clusters, we obtained an inflammation-related dataset from GSEA and applied gene set scoring (**Figure 7B**). The results indicated that DNTs could be divided into two categories based on inflammatory phenotype, aligning well with the observed clustering on the UMAP plot. Therefore, we designated the DNT cluster with high inflammation phenotype scores as Kill+ and the cluster with low inflammation phenotype scores as Kill-(the Kill- group encompasses both anti-inflammatory and undifferentiated states, but due to the low cell numbers, these were combined into a single non-proinflammatory category for simplicity).

To validate our cell subcluster definitions, we obtained inflammation and cytotoxicity-related pathway gene sets from GSEA, including IL-6, NF-κB, TNF-α, and other inflammatory pathways (**Figure 7C**). The results showed that Kill+ DNT cells had higher scores (activation levels) in these inflammatory pathways compared to Kill- DNT cells. Interestingly, when analyzing the proportional changes between Kill+ and Kill- DNT phenotypes, we found that the trend of Kill- DNTs matched the overall trend of DNTs we had previously observed, while Kill+ DNTs exhibited an opposite trend (**Figure 7D**). Meanwhile, by using differential analysis we observed that TMSB10 seemed to be mainly expressed in Kill+ DNT, while it was only weakly positive or not expressed in KILL-DNT (**Supplementary Figure S1**).

Transcription factor analysis also revealed that active-state DNTs had higher transcription factor activity, particularly in cytotoxicity-related factors, compared to resting-state DNTs (**Figure 7E**). Meanwhile, resting-state DNTs displayed a dual expression of both pro-inflammatory and anti-inflammatory transcription factors. This duality may be due to the short modeling period used in our study, as the formation of anti-inflammatory DNTs may require a longer timeframe to manifest fully.

Pseudotime analysis further suggested that Kill- DNTs might serve as precursors to Kill+ DNTs, transitioning from a resting (Kill-) to an active (Kill+) state during IS as they become activated. HLJD treatment appeared to inhibit this transition to a more active state, reducing the migration of Kill+ DNTs into the brain and thereby potentially mitigating inflammation-related damage (**Figure 7F**). To minimize errors introduced by the algorithm, we proceeded with pseudotime analysis using the Monocle3 algorithm. The results were consistent with those obtained from Monocle2, suggesting that DNTs may transition from Kill- DNTs to Kill+ DNTs (**Figures 7G, H**).

These findings underscore the complex role of DNTs in IS, highlighting HLJD’s potential in modulating these cells to reduce inflammatory damage.

### 3.8 Active DNT subtypes exhibit inflammatory and proliferative signatures in ischemic stroke

**Figure 8:**
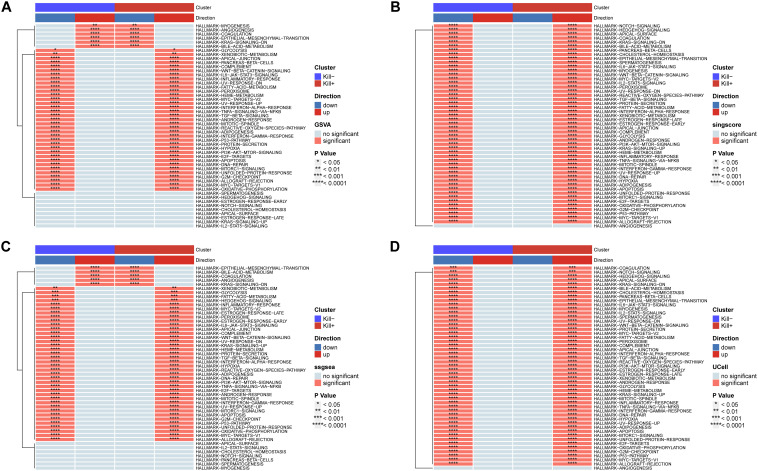
DNT HallMarker gene set scoring. **(A)** Scoring was given by GSVA. **(B)** Scoring was given by singlescore. **(C)** Scoring was given by ssgsea. **(D)** Scoring was given by UCell.

Finally, we conducted a functional evaluation of these two DNT subtypes using UCell, ssGSEA, GSVA, and singlescore, selecting 50 hallmark gene sets from the GSEA database for functional scoring. The results indicated that the active-state DNTs predominantly exhibited an activation-biased phenotype (**Figure 8**). Consistent with our previous findings, the inflammatory pathways in these cells showed marked activation. Furthermore, the activation of E2F, G2M, and NOTCH signaling pathways suggests that active-state DNTs may be in a proliferative state.

### 3.9 Kill+ DNTs exhibit stronger cytotoxic interactions, confirming their pro-inflammatory role

**Figure 9:**
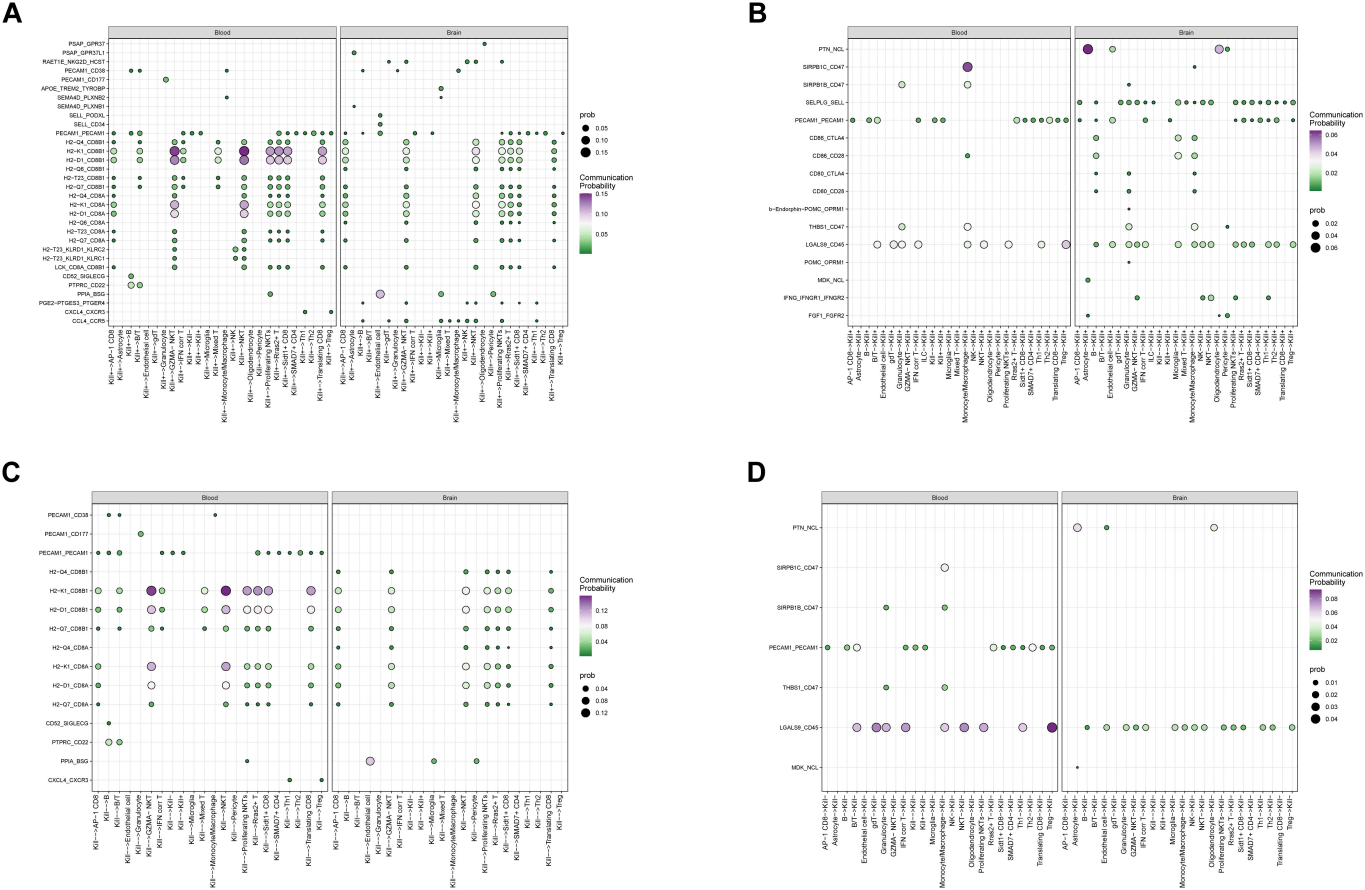
Cell communication relationship analysis. **(A)** kill+ as an outgoing cell communication analysis. **(B)** kill+ as receiving cell communication analysis. **(C)** kill- Cell communication analysis as the emitting end **(D)** kill- Cell communication analysis as receiver.

To explore the mechanisms by which these two DNT subtypes function, we conducted cell-cell communication analysis (**Figures 9A–D**, **Supplementary Figure S2**). Consistent with earlier observations, Kill+ DNTs exhibited more frequent and stronger interactions compared to Kill- DNTs, which showed fewer and weaker interactions. As signal recipients, both DNT subtypes activated the LGALS9_CD45 interaction pathway, with Kill- DNTs demonstrating significantly stronger interaction intensity and a higher proportion of interacting cells than Kill+ DNTs. In T cells, LGALS9 binds to CD45, altering its spatial conformation or inhibiting its phosphatase activity. This process reduces TCR signaling, suppressing T cell function, limiting overactivation, and preventing uncontrolled inflammatory responses. When acting as signal transmitters, Kill+ DNTs predominantly engaged MHC I pathways, such as H2-K1_CD8B1 and H2-D1_CD8B1. Kill+ DNTs showed significantly stronger interaction intensity and a higher proportion of interacting cells compared to Kill- DNTs. These pathways enhance the cytotoxic functions of CD8+ T cells, facilitating the secretion of effector cytokines such as IFN-γ and TNF-α, as well as the release of granzyme and perforin, which directly kill target cells. The target cells for these interactions were primarily CD8+ T cells and NKT cells, consistent with the expected receptor cell types for this pathway. These findings further validate our hypothesis that Kill+ DNTs exhibit a stronger association with cytotoxic and pro-inflammatory functions compared to Kill- DNTs. Additionally, the similarity in cellular interactions between Kill- DNTs and Kill+ DNTs provides evidence supporting the possibility that Kill- DNTs may transition into Kill+ DNTs.

## 4 Discussion

The role of DNT cells in ischemic stroke (IS) remains underexplored, and their specific functions are still not fully understood. Some studies suggest that DNTs can activate microglia, the brain’s resident myeloid immune cells, thereby promoting neuroinflammation and neurodamage (8). Conversely, another study posits that DNTs can induce peripheral myeloid cells to adopt an anti-inflammatory phenotype, leading to neuroprotection and inflammation suppression (9). Similarly, DNTs appear to exhibit differing functional profiles across various research fields and among different investigators, displaying both pro-inflammatory and anti-inflammatory, protective phenotypes (20, 21). This intriguing variability motivated us to pursue further investigation into the role of DNTs in IS.

Our study has several strengths:

1. This is the first study to simultaneously investigate the roles of DNT cells in both the brain and peripheral blood during ischemic stroke (IS) using single-cell transcriptomics. Single-cell transcriptomics, as a high-throughput sequencing approach at the single-cell level, is particularly suited to exploring such cellular heterogeneity. By using individual cells as the primary unit of analysis, single-cell transcriptomics provides a powerful tool to investigate complex cellular diversity, offering valuable insights for advancing our understanding of DNT functionality.
2. Our research focuses on the role of DNT cells during the acute phase of IS, rather than the subacute or chronic phases reported in previous studies. This partially explains the inconsistent findings regarding DNT functionality in prior research.
3. Additionally, our study demonstrates that HLJD may exert therapeutic effects on IS by modulating DNT cells. This aspect has been relatively overlooked in previous TCM-related studies and provides new perspectives for the application of traditional Chinese medicine in the treatment of IS.

In our mouse data, both peripheral blood and brain samples showed an increase in DNT cells following stroke, consistent with previous reports. Some studies have indicated that exposure to inflammatory CNS antigens can lead to the *in situ* proliferation and expansion of T cells, providing an additional explanation for the accumulation of DNT cells in the ischemic brain beyond their migration from peripheral sources (22, 23). Interestingly, after administering HLJD, we observed a decrease in DNT cells in the blood and an increase in the brain. At the same time, pharmacodynamic evaluations indicated significant improvement in stroke symptoms in HLJD-treated mice. These findings led us to consider two possibilities: (1) There may be functionally distinct subsets within the DNT population; (2) HLJD may promote the chemotaxis of DNTs toward the brain or stimulate *in situ* proliferation of DNTs within the brain.

This aligns with the notion that traditional Chinese medicine (TCM) formulas, as multi-target and multi-pathway interventions, may modulate disease through diverse mechanisms simultaneously. Previous studies on HLJD support this multifaceted therapeutic potential.

The multi directional nature of DNT cells suggests a level of functional complexity, indicating that DNTs may not represent a single, uniform phenotype. Similar to CD4+ and CD8+ subtypes within T cells, or M1 and M2 phenotypes within monocytes/macrophages, DNTs might be divided into distinct subtypes, each with unique functions.

While our findings shed light on the role of DNTs in IS and explore the regulatory effects of HLJD, several critical questions remain unresolved. Firstly, we performed only a subpopulation analysis of DNTs; however, the inherent dropout issue in single-cell transcriptomics limits our ability to capture the complete transcriptome for each cell, posing challenges in identifying reliable biomarkers for Kill+ and Kill- DNTs. Secondly, the functions of these two DNT subtypes at different time points in IS may represent an important area for further study, as their roles may vary across stages of disease progression. Lastly, due to the lack of spatial information and cellular positioning, we cannot fully assess the interactions between DNTs and other cell types—a crucial aspect that we hope to explore in future studies when resources and technology allow.

## 5 Conclusion

Our findings indicate that in the pathogenesis of ischemic stroke (IS), there may be two functionally distinct DNT subtypes: an activated, cytotoxic DNT and a resting, immunosuppressive DNT. Both appear to play crucial roles in the pathological progression of IS. Treatment with HLJD may reduce the population of cytotoxic DNTs while increasing immunosuppressive DNTs and modifying their immune infiltration patterns, thereby helping to alleviate brain damage associated with IS.

## Data Availability

The original contributions presented in the study are publicly available. This data can be found here: https://ngdc.cncb.ac.cn/gsa/search?searchTerm=CRA020927.
