# Supplementary figures and images for: Modulation of double-negative T cells by Huang-Lian-Jie-Du Decoction attenuates neuroinflammation in ischemic stroke: insights from single-cell transcriptomics
Source: Front Immunol. 2025 Feb 13;16:1537277. doi: 10.3389/fimmu.2025.1537277 (PMC11865039; doi:10.3389/fimmu.2025.1537277)

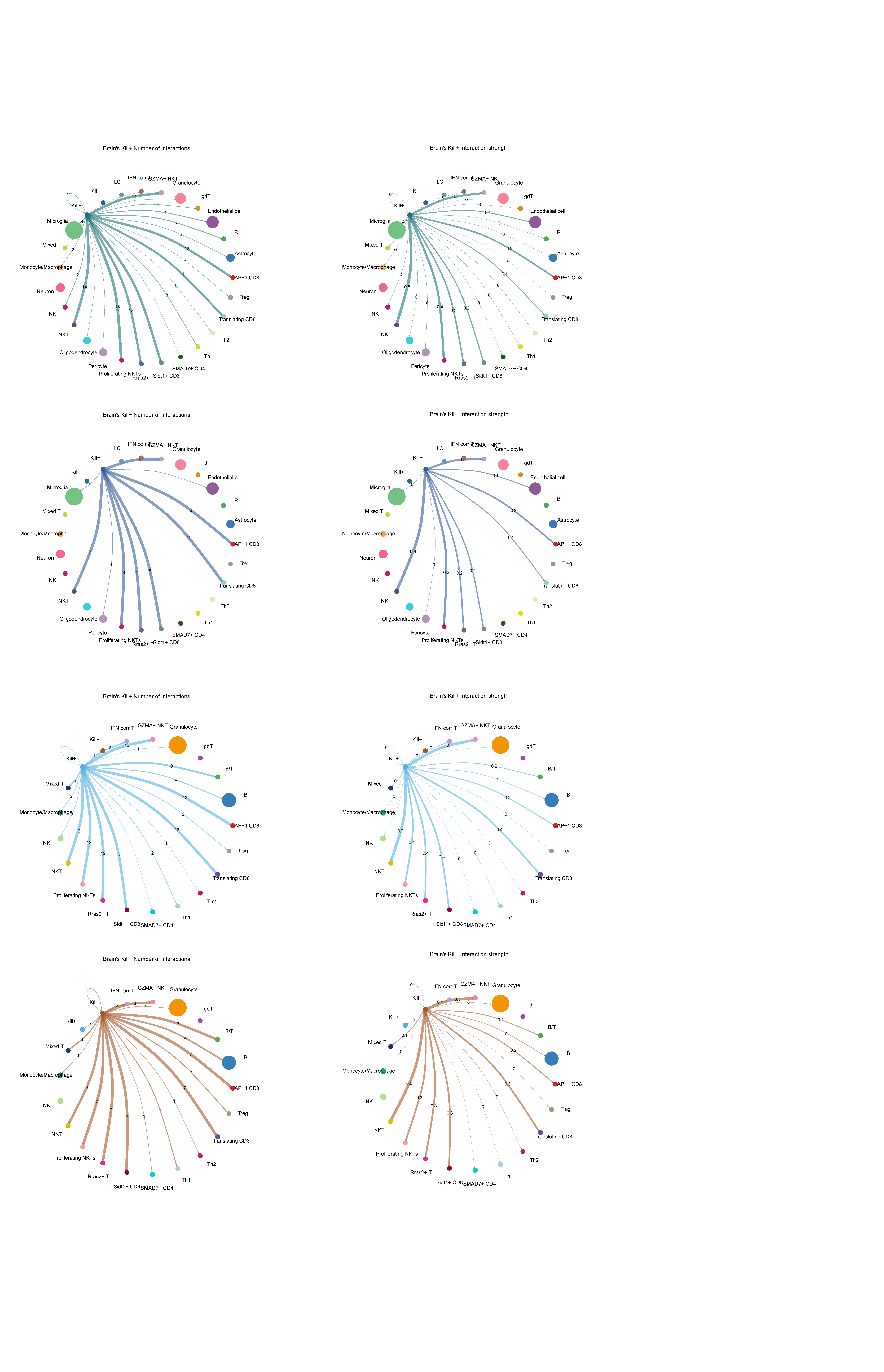

Supplement: Supplementary file 1 [file Image1.tif]

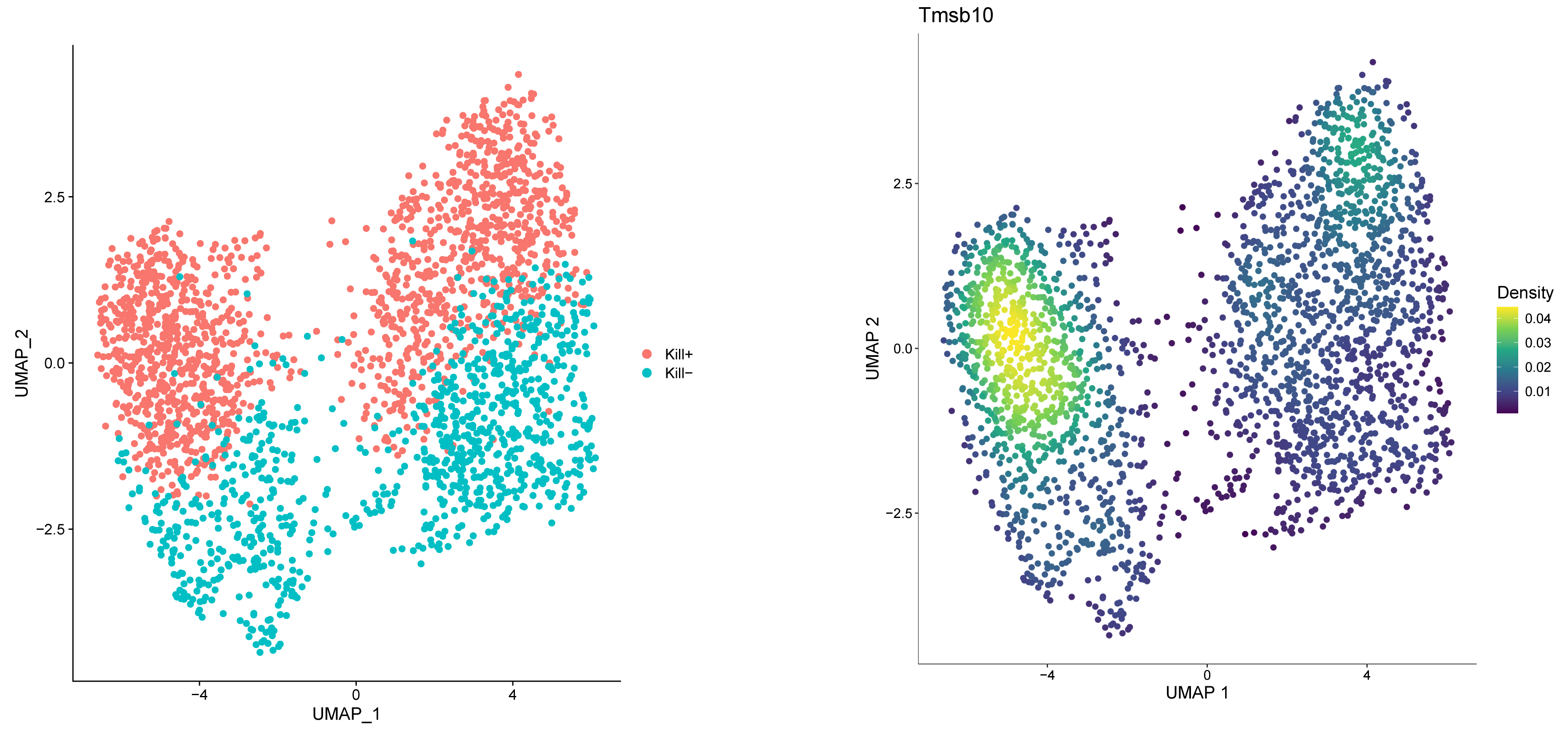

Supplement: Supplementary file 2 [file Image2.tif]
